# Laser Scanning Morphometric Measurements of the Main Orbital Communications in Dry Human Skulls

**DOI:** 10.3390/diagnostics14192168

**Published:** 2024-09-29

**Authors:** Ruxandra Coroleucă, Florin Mihail Filipoiu, Alina Popa Cherecheanu, Mihaly Enyedi, Radu Bucșan, Mihai Bostan, Ciprian-Andrei Coroleucă, Lidia Ladea, Daniela Vrînceanu, Oriana Elena Moraru, Raluca Iancu

**Affiliations:** 1 Ophthalmology Department, “Carol Davila” University of Medicine and Pharmacy, 050474 Bucharest, Romania; alina.cherecheanu@umfcd.ro (A.P.C.); radu-gheorghe.bucsan@drd.umfcd.ro (R.B.); mihai.bostan@drd.umfcd.ro (M.B.); raluca.iancu@umfcd.ro (R.I.); 2 Bucharest Emergency University Hospital, 050098 Bucharest, Romania; daniela.vrinceanu@umfcd.ro; 3 Anatomy Department, “Carol Davila” University of Medicine and Pharmacy, 050474 Bucharest, Romania; florin.filipoiu@umfcd.ro (F.M.F.); mihaly.enyedi@umfcd.ro (M.E.); 4 Obstetrics-Gynecology Department, “Carol Davila” University of Medicine and Pharmacy, 050474 Bucharest, Romania; ciprian.coroleuca@umfcd.ro; 5 Doctoral School, “Carol Davila” University of Medicine and Pharmacy, 050474 Bucharest, Romania; lidia.ladea@drd.umfcd.ro; 6 ENT Department, “Carol Davila” University of Medicine and Pharmacy, 050474 Bucharest, Romania; 7 Discipline of Cardiovascular Surgery, “Carol Davila” University of Medicine and Pharmacy, 050474 Bucharest, Romania; oriana-elena.moraru@umfcd.ro; 8 Emergency Clinical Hospital “Prof. Dr. Agrippa Ionescu”, 011356 Bucharest, Romania

**Keywords:** craniometry, dry skull, laser scanning measurement, anthropometry, orbital foramen, superior orbital fissure, inferior orbital fissure

## Abstract

Background and Objectives: This research investigated the morphometric dimensions of the optic foramen (OF), superior orbital fissure (SOF) and inferior orbital fissure (IOF), using indirect measurement techniques such as laser scanning, making it likely the first study of its kind. This study aimed to identify the morphometric variability of the main orbit communications and to highlight the differences between genders. Materials and Methods: The anthropometric study was conducted on sixty dry skulls (120 orbits) of adults aged between 20 and 70 years. Measurements of orbital communications were made using the RS6 laser scanner. The orbital parameters that were investigated are as follows: length and width of the SOF and IOF, and height and width of the OF. Results: In males, the average height of the OF was 8.27 mm and 8.13 mm in females, while the average width of the OF was 6.34 mm in males and 5.83 mm in females. The SOF average length was 21.09 mm in males and 17.58 mm in females. The widths of the SOF in the three thirds (anterior, middle and posterior) in males were 5.14/4.77/7.11 mm and 2.28/3.48/5.80 mm in females. The average length of the IOF was 33.05 mm in males and 32.30 mm in females. The widths of the IOF in the three thirds (anterior, middle and posterior) were 5.61/3.92/4.70 mm in males and 7.24/4.68/4.08 mm in females. Conclusions: The OF height and width were higher in males compared to females. The SOF length and width were higher in males compared to females. The IOF length was higher in males for the right orbit and higher in females for the left orbit. The IOF width for both orbits was higher in females in the anterior and middle third, and higher for males in the posterior third. Evaluation of dry skulls using laser scanning is reliable and recommended for data accuracy. Laser scanning can become a usable method for all indented and hard-to-reach regions of the cranial skeleton.

## 1. Introduction

The orbit presents a complex anatomy. The orbital cavity serves as a protective barrier for the eyeball, enabling essential functions such as connecting with the surrounding world, adapting to the environment and maintaining spatial orientation [1,2]. In this anatomical bone structure of relatively small dimensions, many anatomical elements are condensed and establish close relationships that are particularly important for the evaluation, diagnosis and treatment of orbital pathologies [1,2].

The orbital cavity communicates with neighboring structures via foramina and fissures. There are three main pathways through which the orbit communicates with adjacent regions: the optic canal, the superior orbital fissure and the inferior orbital fissure [3,4]. Through these openings, the orbit connects with the middle cranial fossa, the pterygopalatine fossa and the infratemporal fossa [3,4]. Communication between the orbit and adjacent areas are particularly important in ocular and orbital pathology, as these pathways serve as routes for the spread of infections and areas of low resistance for tumor spread [4].

The optic canal, situated at the posterior end of the orbital roof at the orbital apex, is a bony passage that connects the orbit to the middle cranial fossa. On the lateral aspect of the sphenoid bone, we can identify the two roots of the small wing that define the optic canal’s boundaries between them. Thus, the optic canal is bounded medially by the body of the sphenoid bone, superiorly by the anterior root of the lesser wing of the sphenoid bone and infero-medially by the posterior root of the lesser wing of the sphenoid bone (known as the optic bridge) [1,2,3]. The optic canal has three parts: the first part is represented by the orbital opening, the second part is represented by the optic canal itself and the third part is its opening into the middle cranial fossa. The first part is known as the optic foramen (OF). The second part has a direction from the supero-medial to the infero-lateral, and the third part is bounded superiorly by the falciform fold and laterally by the anterior clinoid process [1,2]. The optic nerve and its meningeal sheaths, together with the ophthalmic artery which is located inferolaterally to the nerve, pass through the optic canal [1].

The superior orbital fissure (SOF) is a bony cleft that connects the orbital apex with the middle cranial fossa. The SOF lies within the sphenoid bone and is bounded medially by the body and the lesser wing of the sphenoid, and laterally by the greater wing of the sphenoid. This fissure resembles a comma, with a more rounded infero-medial portion and a narrower and more elongated supero-lateral portion [1,2,3,4]. Therefore, we can characterize the four sides of the superior orbital fissure, delineated by bony structures, as follows. The medial edge of the SOF is less defined and is represented inferiorly by the body of the sphenoid bone and superiorly by a bone segment (optic strut) that connects the body of the sphenoid to its lesser wing [1,2,3]. The upper edge of the SOF is bounded medially to laterally by the optic strut, the base of the anterior clinoid process and the lower surface of the lesser wing of the sphenoid bone. The lateral border of the SOF is bounded by the greater wing of the sphenoid bone. The lower border of the SOF is formed laterally by the greater wing of the sphenoid bone and medially by the junction of the greater wing with the body of the sphenoid. Thus, the SOF is a bony slit located at the junction of the greater and lesser wings of the sphenoid bone [1]. The vasculo-nervous elements that cross the SOF are as follows: the orbitomeningeal arteries; the veins of the dura mater; the superior ophthalmic vein; the inferior ophthalmic vein; the trochlear nerve (IV); the frontal, lacrimal and nasociliary branches of ophthalmic nerve (V); the superior and inferior branches of the oculomotor nerve (III); and the abducens nerve (VI) [4].

The inferior orbital fissure (IOF) separates the lateral orbital wall from the inferior orbital wall in the posterior part of the orbit. The IOF is situated between the greater wing of the sphenoid bone on the lateral side and the orbital process of the maxillary and palatine bones on the medial side, with the anterior boundary being formed by the zygomatic bone. The IOF connects the floor of the orbit with the following: the temporal fossa (through the anterior third), the infratemporal fossa (through the middle third) and the pterygopalatine fossa (through its posterior third) [1,2].

Details related to the morphology and morphometry of the orbital foramina and fissures provide important information that can be used in various fields: in anthropology (establishing the morphological and morphometric characteristics of the orbits and comparing them with the classic population values); in forensic medicine (by highlighting gender differences in orbital parameters); and in clinical practice, where morphometric values and the presence of anatomical landmarks can be used in ophthalmology, endocrinology, otorhinolaryngology, neurosurgery or plastic and reconstructive surgery [5,6,7,8].

Traditional craniometry involves taking direct measurements of the skull using specialized tools and anatomical landmarks. Since the early publications on morphometrics in the late 1970s, significant technological advancements have introduced various alternative methods for gathering morphological data. Recently, this field has evolved with the incorporation of digital techniques. Modern imaging technologies now facilitate the creation of 2D and 3D images, allowing for metric analyses in a virtual setting. Additionally, craniometric measurements can be obtained from 3D models generated through various methods, including laser scanning.

Three-dimensional laser surface scanners have gained popularity in skull morphometry research due to their ability to capture complex shapes and detailed surface features efficiently and accurately. While these scanners offer significant advantages, it’s essential to ensure that their accuracy meets or exceeds that of existing methods [9].

Laser scanning of skulls prove to be an innovative and usable method. Park’s 2006 study concluded that handheld 3D laser scanning, when combined with the precise location of points on a 3D scanned skull, is a highly effective technique for craniometry. Their method showed excellent intra- and inter-rater reliability, indicating that the measurements are consistent and reproducible across observers and studies. Furthermore, the study concluded that the accuracy and quality of craniometric data obtained by this 3D scanning technique is comparable to that derived from traditional caliper measurements. This suggests that hand-held 3D laser scanning could serve as a viable alternative to conventional methods in craniometric analyses [10].

The findings from Toneva’s study regarding the accuracy of linear craniometric measurements, obtained from laser scanning, indicated that a significant majority of digital measurements were comparable to direct measurements, with 96% showing deviations of less than 2 mm and 67.6% within 1 mm. Also, the authors emphasized the fact that some digital measurements, especially those related to landmarks on bone margins, consistently showed an overestimation when compared to direct measurements. These differences underscore the necessity of choosing measurement techniques carefully based on the specific anatomical landmarks [11].

Measuring the deep foramina and fissures prove challenging with the classical methods used in craniometry, and we found laser scanning to be the most appropriate method. The limitation of the classical methods consists of the impossibility in using the instruments in a narrow bony space such as the orbital apex.

Data in the literature regarding the measurements of the orbital foramina and fissures come from studies using direct measurement of these fissures through the endocranial or infratemporal approach and tomographic scanning with orbital reconstruction [3,5,6,7,8,12,13]. Our study evaluates the dimensions of the orbital foramina and fissures from the orbital perspective using laser scanning. To our knowledge, this is the first study of its kind carried out on a Romanian or international population group.

## 2. Materials and Methods

This anthropometric study was performed on dry skulls from the osteological collection of the Anatomy Department of the “Carol Davila” University of Medicine and Pharmacy in Bucharest. For this purpose, 60 dry skulls (120 orbits) of adults aged between 20 and 70 years were analyzed. The sex and age were noted for each individual. Skulls with malformations, fractures or incomplete orbits were excluded from the morphometric study.

**Figure 1:**
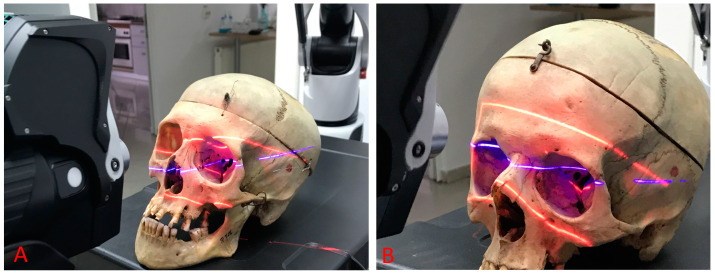
(**A**,**B**) RS6 laser scanning of the orbital foramen and fissures.

The scans were carried out by the Hexagon Manufacturing Intelligence Company (Bucharest), specialized in measurements and metrology. For scanning the orbits, we used the RS6 laser scanner (Hexagon AB, Stockholm, Sweden) placed on a measuring arm with multiple articulated joints, allowing for easy handling of the scanner and to maximize accuracy. The modular arm joint, together with the RS6 scanner, made measurements flexible, enabled scanning in multiple planes and ensured fast and safe operation (Figure 1). The main technical characteristics of the RS6 laser scanner, according to the device’s technical specifications, are as follows: high precision of measurements (accuracy of 0.026 mm), maximum speed of point collection (up to 1.2 million points/second), maximum number of scanned points per line (4000), maximum frequency of scanning lines (300 Hertz), length of scanning line (middle)–150 mm, average scanning distance–165 ± 50 mm, minimum distance between scanned points (line)–0.027 mm and laser class–2 M. The RS6 laser scanner provided the capability to gather extensive quantities of data without compromising the data quality. Approximately 20 million points were scanned for each orbit.

**Figure 2:**
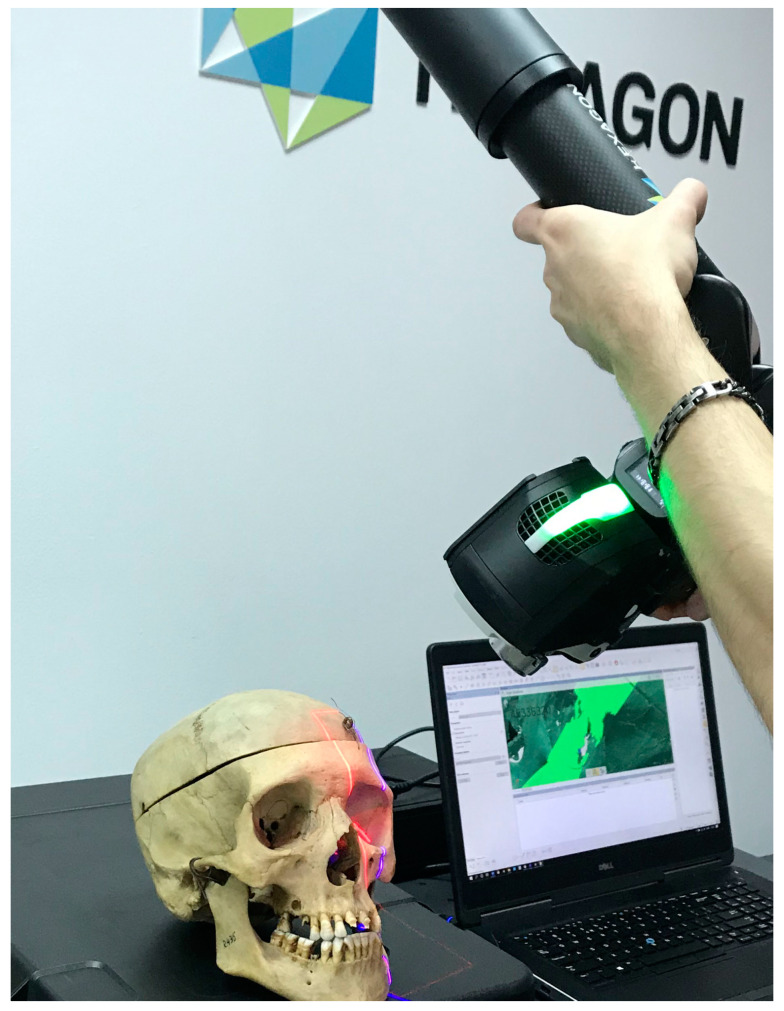
Scanning the orbital foramina using the laser and viewing the scanned area in real time.

**Figure 3:**
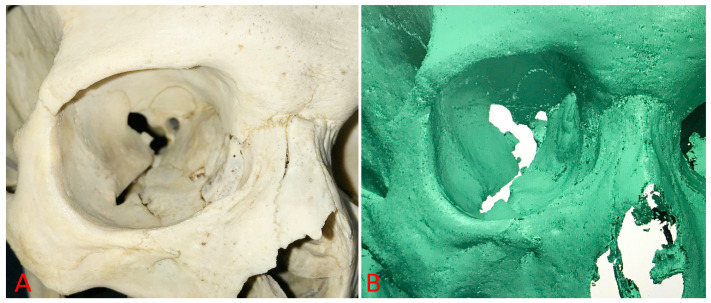
Original skull (**A**) and the result of the laser scanner (**B**).

The RS6 laser scanner was connected to software that, with the help of the Polyworks Inspector Premium program version IR11.2 developed by the InnovMetric Company, Quebec, QC, Canada), allowed for real-time visualization of the performed scans (Figure 2 and Figure 3). Polyworks Inspector Premium program version IR 11.2 is a three-dimensional analysis software solution used for making orbital measurements. All of these measurements were made after three-dimensional visualization of the orbital relations.

The measurements made at the orbital apex concern the following foramina:

- SOF length (the distance from the anterior edge to the posterior edge of the SOF);
- SOF width (the SOF was divided into three equal parts; each part was measured at its midpoint, resulting in three diameters);
- IOF length (the distance from the anterior point to the posterior point of the IOF);
- IOF width (the IOF was divided into three equal parts; each part was measured at its midpoint, resulting in three diameters);
- Orbital OF height (the distance from the highest point to the lowest point of the orbital opening);
- Orbital OF width (the distance from the most medial point to the most lateral point of the orbital opening).

The collected data were entered in the Microsoft Office Excel version 15.0.5589.1000 program and later converted into an SPSS format. Statistical analysis of the data was carried out using the SPSS 23.0 program. The results of the orbital foramina measurements are provided as the mean, standard deviation (SD) and minimum and maximum values.

## 3. Results

### 3.1. Measurements of the Orbital OF

The male OF height was 8.32 ± 0.49 mm for the right orbit, 8.22 ± 0.25 mm for the left orbit and 8.27 ± 0.24 mm (minimum 7.40–maximum 9.16 mm) for both orbits. The female OF height was 7.86 ± 0.44 mm for the right orbit, 8.26 ± 0.89 mm for the left orbit and 8.13 ± 0.62 mm (minimum 6.27–maximum 8.60 mm) for both orbits (Table 1).

For males, the OF width was 6.51 ± 0.73 mm for the right orbit, 6.16 ± 0.52 mm for the left orbit and 6.34 ± 0.57 mm (minimum 5.01–maximum 8.10 mm) for both orbits. For females, the OF width was 5.68 ± 0.50 mm for the right orbit, 5.97 ± 0.94 mm for the left orbit and 5.83 ± 0.67 mm (minimum 4.51–maximum 7.41 mm) for both orbits (Table 2).

The OF height and width of the right orbit were significantly higher in males compared to females (*p* < 0.001). The OF height and width of the left orbit were insignificantly higher in males (*p* = 0.367, *p* = 0.248).

### 3.2. Measurements of the SOF

The male SOF length was 19.70 mm for the right orbit, 21.00 ± 2.42 mm for the left orbit and 21.09 ± 2.57 mm for both orbits (minimum 16.76–maximum 27.60 mm). The female SOF length was 17.91 ± 1.80 mm for the right orbit, 17.25 ± 1.63 mm for the left orbit and 17.58 ± 1.70 mm for both orbits (minimum 15.16–maximum 21.12 mm) (Table 3). The superior orbital fissure length was significantly higher for males compared to females (*p* < 0.001).

The superior orbital fissure width in the anterior third for males at the right orbit had a median of 5.45 mm, a mean of 5.13 ± 2.18 mm for the left orbit and a mean of 5.14 ± 1.99 mm for both orbits. For the women, the SOF width in the anterior third at the right orbit had a median of 2.42 mm, a mean of 1.91 ± 0.59 mm for the left orbit and a mean of 2.28 ± 0.61 mm for both orbits (Table 4).

The superior orbital fissure width in the middle third for males was 4.90 mm for the right orbit, 4.51 ± 0.80 mm for the left orbit and 4.77 ± 0.59 mm for both orbits. For the women, the SOF width in the middle third was 3.42 ± 0.86 mm for the right orbit, 3.55 ± 0.93 mm for the left orbit and 3.48 ± 0.85 mm for both orbits (Table 4).

The male SOF width in the posterior third was 7.68 ± 1.49 mm for the right orbit, 6.12 mm for the left orbit and 7.11 mm for both orbits. The female SOF width in the posterior third was 5.91 ± 0.65 for the right orbit, 5.69 ± 0.71 mm for the left orbit and 5.80 ± 0.65 mm for both orbits (Table 4).

The SOF length in both orbits was significantly higher for males compared to females (*p* < 0.001). The SOF width in both orbits in all three thirds was significantly higher in males compared to females (*p* < 0.001).

### 3.3. Measurements of the IOF

The male IOF length was 33.61 mm for the right orbit, 33.31 ± 3.51 mm for the left orbit and 33.05 mm for both orbits (minimum 27.30–maximum 40.20 mm). The female IOF length was 31.61 mm for the right orbit, 34.17 ± 3.22 mm for the left orbit and 32.30 mm for both orbits (minimum 28.77–maximum 39.27 mm) (Table 5). The right orbit IOF length was significantly higher for males compared to females (*p* < 0.001). The left orbit IOF length was insignificantly lower for males (*p* = 0.391).

The inferior orbital fissure width in the anterior third for males was 5.54 ± 1.23 mm for the right orbit, 5.68 ± 1.35 mm for the left orbit and 5.61 ± 1.28 mm for both orbits. For the women, the IOF width in the anterior third was 6.97 ± 1.44 mm in right orbit, 7.51 ± 1.58 mm for the left orbit and 7.24 ± 1.46 mm for both orbits (Table 6).

The inferior orbital fissure width in the middle third for males was 3.80 mm for the right orbit, 3.94 ± 0.96 mm for the left one and 3.92 ± 0.87 mm for both orbits. For the women, the IOF width in the middle third was 4.36 mm for the right orbit, 5.20 ± 1.59 mm for the left one and 4.68 ± 1.0 mm for both orbits (Table 6).

The inferior orbital fissure width in the posterior third of the right orbit was 5.67 mm for males and 3.59 mm for females. IOF width in the posterior third of the left orbit was 4.43 ± 1.35 mm for males and 4.06 ± 1.80 mm for females. In both orbits, the IOF width in the posterior third was 4.70 ± 1.21 mm for males and 4.08 ± 1.55 mm for females (Table 6).

The IOF width in both orbits in the anterior third was significantly higher for males compared to females (*p* < 0.001). The IOF width in both orbits in the middle third was higher for males compared to females (right orbit *p* = 0.120, left orbit *p* < 0.001). The IOF width in both orbits in the posterior third was higher for males compared to females (right orbit *p* = 0.069, left orbit *p* = 0.104).

## 4. Discussion

The orbits represent important anatomical landmarks at the skull level. The morphology and morphometry of the orbit have become objects of forensic interest for more than 100 years [6]. In addition to their importance in fields such as anthropology, legal medicine and forensics, orbital morphometry and morphology have an obvious importance in clinical practice, where these variables play an important role in orbital reconstruction and the restoration of full functionality [7,8].

The results of this study regarding the height and width of the OF were 8.27/6.34 mm for males and 8.13/5.83 mm for females, with higher values for males compared to females. Analyzing these values, we can conclude that the OF has an oval shape, with the vertical diameter larger than the horizontal one. These data are consistent with the results obtained by other authors in their studies, although they used other measurement methods [12,13,14,15,16,17]. Comparing our results with previous studies, we can notice some differences, particularly in the height of the OF as shown in Table 7. The data concerning the height and width of the OF in the current study were acquired through laser scanning of the OF and subsequent measurements from an orbital perspective. Although we did not come across similar studies in the literature, comparing our findings with data from studies that used CT scanning or direct measurement methods reveals significant variance. Most authors report a height between 5.35–5.7 mm and a width between 4.74–4.91 mm on direct measurements and even smaller values on CT scans (height 4.12–5.01 mm and width 2.98–5.66 mm) [12,13,14,15,16]. The optic foramen is a deeply situated orbital landmark that poses challenges for direct measurement. The methodology employed in the current study differs from that utilized by other authors. One possible explanation for the variances observed in our study could be attributed to the methodology employed, where laser measurements allowed us to assess the OF within the orbit’s opening rather than at its entrance into the optic canal.

The morphometric differences between sexes regarding the OF, according to the present study, show that the right OF height is significantly higher for males compared to females (*p* < 0.001). The left OF height is insignificantly higher for males (*p* = 0.367). The same statistical significance is attributed to the width of the OF: the right OF is significantly greater for males compared to females (*p* < 0.001), while the left OF width is greater for males, but insignificant (*p* = 0.248).

In present study, the average SOF length for males was 21.09 mm, and for females 17.25 mm, and this difference is significantly higher for males compared to females (*p* < 0.001). Govsa et al. reported similar SOF lengths in dry skulls, measuring 20.8 ± 3.9 mm on the right and 20.1 ± 3.8 mm on the left side [18]. Conversely, Shukla et al. reported lower values of approximately 14 mm (left SOF 1.39 ± 0.21 cm and right SOF 1.4 ± 0.24 cm) for the Indian population [19]. Berlis et al. measured the SOF on dry skulls and performed CT scans of the same skulls. They reported similar lengths of 20.05 mm for both methods and concluded that high resolution CT, with 1 mm sections, provided precise measurements that differed little from direct measurements on skulls. However, it should be taken into account that the CT measurements must be made in certain incidents so that the foramina lies perpendicular to the scanline [16]. On the other hand, Patel et al., in their study on dry skulls and patients’ CT scans, obtained different values between the direct measurement and the CT scans. The average SOF length for direct measurement at the cranial level was approximately 16 mm (15.93 mm for the right SOF, 16.18 mm for the left SOF) and on the CT measurement it was approximately 11 mm (10.90 mm for the right SOF and 10.92 mm for the left SOF) [3].

In our study, the SOF width was reported to be 5.14 mm in the anterior third, 4.77 mm in the middle third, and 7.11 mm in the posterior third for males, compared to 2.28 mm, 3.48 mm, and 5.80 mm for females. Along its entire length, the SOF was wider for males compared to females, with these differences being statistically significant (*p* < 0.001). In both sexes, the SOF was antero-laterally narrower and became postero-medially wider towards the base. Morphological details of the SOF are of obvious clinical importance due to the variability in the position of the superior ophthalmic vein. The SOF morphology is divided into two categories: category “a”, which has an obvious narrowing in the middle third; and category “b”, which does not present this narrowing [20]. Raymond et al., in their cadaveric study of 100 orbits, demonstrated that the morphological variability of the SOF influences the position of the soft tissues. In type “a”, the superior ophthalmic vein was located typically in the supero-lateral part of the SOF, whereas in type “b”, the superior ophthalmic vein was placed in a low or very low position [20].

Fujiwara et al. reported a SOF width of 3.21 ± 1.07 mm from cadaver study and a width of 3.73 ± 1.64 mm from CT scans, where the SOF width was measured at the level of the optic canal [21]. Other authors reported similar values of the SOF width at the level of the optic canal being approximately 3.7 mm (3.79 mm for men and 3.65 mm for women) [22]. Measurements of the SOF at the optic canal level indicate the width of the SOF at approximately its midpoint. Direct comparison between data from the literature and the findings of our study is challenging due to variations in measurement methods. However, our findings show that the width of the SOF in the middle third was 4.77 mm for men and 3.48 mm for women, values that closely align with those reported in the literature [21,22].

Our study indicates that the SOF width of women is smaller along its entire length. Upon comparing the measurements of the right and left orbits, our data show that they are similar or comparable in size. Understanding the width of the SOF is crucial in clinical settings as a narrower SOF can lead to the compression of important structures passing through it, potentially resulting in conditions such as superior orbital fissure syndrome. For instance, a smaller SOF width can result in the compression of the cranial nerves (such as the oculomotor, trochlear, and abducens nerves and the ophthalmic division of the trigeminal nerve), leading to various neurological symptoms like ophthalmoplegia, ptosis, fixed dilated pupils and facial numbness [23,24,25]. This emphasizes the significance of assessing the SOF width in clinical evaluations to understand potential risk factors and implications for patients [21]. In trauma cases, careful manipulation of anatomical structures is necessary to avoid exacerbating nerve compression or causing additional damage to structures passing through the fissure.

Understanding morphology, morphometry and the surrounding structures of the IOF is essential in clinical practice, especially in cases involving trauma, tumors or infections in the maxillofacial region. The IOF’s location in the orbital floor, near critical areas such as the superior orbital fissure, foramen rotundum, pterygopalatine fossa, infratemporal fossa and temporal fossa, underscores its anatomical significance [26,27,28,29,30,31,32].

The significant level of symmetry noted in the IOF morphology across the 60 skulls we examined offers valuable insights into the anatomical uniformity within this region. Approximately 90% of the skulls exhibited symmetrical features between the right and left orbits. The classification system proposed by Ozer et al., outlining eight distinct morphological types for IOF, offers valuable insights into the variations seen in this anatomical feature. Among these types, type 1 stands out as the most commonly encountered variation (42%). In type 1, the IOF is distinguished by having its widest part situated laterally and narrowing towards the medial part [33]. Our study’s findings align with Ozer’s classification, with type 1 being the most prevalent morphological type observed, constituting around 40% of the studied skulls. The average IOF width in the three thirds from anterior to posterior was 5.61/3.92/4.70 mm for men and 7.24/4.68/4.08 mm for women. Based on Ozer’s morphological classification and our data analysis, it is evident that men often demonstrate type I IOF morphology, whereas women tend to show type V characteristics (a triangular shape with a wider anterior part that narrows postero-medially) [33].

De Battista’s study on 50 dry skulls found an average IOF length of 29.1 mm, with significant variability ranging from 23 mm to 35 mm. In contrast, our study recorded an average IOF length of 32.6 mm (33.5 mm for men and 32.3 mm for women), surpassing De Battista’s average measurement [34].

The difference in segmenting the IOF between our study and De Battista’s study introduces a methodological inconsistency that hinders the direct comparison of results, especially in measuring the width of the IOF. De Battista et al. divided the foramen into three unequal parts, with the medial segment representing the infraorbital canal [34]. These measurements in the three segments were 5 mm, 3.2 mm, and 2.4 mm, respectively [34]. Our study revealed slightly higher values for the width of the IOF segments: 6.42 mm, 4.3 mm and 4.39 mm from the anterior to posterior.

One notable limitation of this study is the challenge in comparing our data with existing literature due to variations in methodologies among different studies, with no directly comparable research found. Another limitation is the relatively small sample size of the dry skulls (60 skulls, 120 orbits) analyzed.

## 5. Conclusions

The OF height and width were higher for males compared to females. The SOF length and width were higher for males compared to females. The IOF length was higher for males for the right orbit and higher for females for the left orbit. The IOF width for both orbits was higher for females in the anterior and middle third, and higher for males in the posterior third.

The evaluation of dry skulls using laser scanning is reliable and recommended for data accuracy. This technique proves particularly effective for capturing detailed measurements in intricate and hard-to-reach regions of the cranial skeleton.

## Figures and Tables

**Table 1:** Descriptive analysis of the OF height, mean ± standard deviation (SD), minimum and maximum values (mm).

OF Height	Males			Females		
Orbit	Right	Left	Both	Right	Left	Both
Mean	8.32	8.22	8.27	7.86	8.26	8.13
SD	0.49	0.35	0.34	0.44	0.89	0.62
Min	7.40	7.80	7.63	6.61	6.27	6.62
Max	9.08	9.16	8.85	8.44	8.60	8.38

**Table 2:** Descriptive analysis of the OF width, mean ± SD, minimum and maximum values (mm).

OF Width	Males			Females		
Orbit	Right	Left	Both	Right	Left	Both
Mean	6.51	6.16	6.34	5.68	5.97	5.83
SD	0.73	0.52	0.57	0.50	0.94	0.67
Min	5.21	5.01	5.11	4.78	4.51	4.65
Max	8.10	6.76	7.23	6.42	7.41	6.92

**Table 3:** Descriptive analysis of the SOF length, mean ± SD, minimum and maximum values (mm).

SOF Length	Males			Females		
Orbit	Right	Left	Both	Right	Left	Both
Mean	19.70	21.00	21.09	17.91	17.25	17.58
SD	-	2.42	2.57	1.80	1.63	1.70
Min	17.39	16.76	17.08	15.16	15.26	15.21
Max	27.60	24.00	25.80	21.12	21.01	21.07

**Table 4:** Descriptive analysis of the SOF width in the anterior, middle and posterior third, mean ± SD.

SOF Width	Males			Females		
Orbit	Right	Left	Both	Right	Left	Both
Mean SOF width ant 1/3	5.45	5.13	5.14	2.42	1.91	2.28
SD	-	2.18	1.99	-	0.59	0.61
Mean SOF width mid 1/3	4.90	4.51	4.77	3.42	3.55	3.48
SD	-	0.80	0.59	0.86	0.93	0.85
Mean SOF width post 1/3	7.68	6.12	7.11	5.91	5.69	5.80
SD	-	1.20	1.08	0.65	0.71	0.65

**Table 5:** Descriptive analysis of the IOF length, mean ± SD, minimum and maximum values (mm).

IOF Length	Males			Females		
Orbit	Right	Left	Both	Right	Left	Both
Mean	33.61	33.31	33.05	31.61	34.17	32.30
SD	-	3.51	-	-	3.22	-
Min	32.90	27.30	30.15	29.67	28.77	30.19
Max	40.20	38.38	39.21	37.34	39.27	38.31

**Table 6:** Descriptive analysis of the IOF width in the anterior, middle and posterior third, mean ± SD.

IOF Width	Males			Females		
Orbit	Right	Left	Both	Right	Left	Both
Mean IOF width ant 1/3	5.54	5.68	5.61	6.97	7.51	7.24
SD	1.23	1.35	1.28	1.44	1.58	1.46
Mean IOF width mid 1/3	3.80	3.94	3.92	4.36	5.20	4.68
SD	-	0.96	0.87	-	1.59	1.07
Mean IOF width post 1/3	5.67	4.43	4.70	3.59	4.06	4.08
SD	-	1.35	1.21	-	1.80	1.55

**Table 7:** Comparison of the measurements from the present study with those from previous studies (OF height and width).

Paramenters (mm)	Radunovic et al. [13], (2019)	Govsa et al. [14], (1992)	Hart et al. [15], (2009)	Kalthur et al. [12], (1992)		Berlis et al. [16], (1992)		Present Study
Measurement	Direct (right/left)	Direct	CT	CT	Direct	CT	Direct	Laser scan (M/F)
OF height	5.43/5.38	5.35	4.90	4.12	5.70	5.01	5.46	8.27/8.13
OF width	4.91/4.86	4.74	4.50	2.98	4.74	5.66	4.75	6.34/5.83

## Data Availability

The datasets used and/or analyzed during the current study are available from the corresponding authors upon reasonable request. The dataset(s) supporting the conclusions of this article is(are) included within the article.

## Abbreviations

The following abbreviations are used in this manuscript:

OF	orbital foramen
SOF	superior orbital fissure
IOF	inferior orbital fissure
SD	standard deviation
CT	computed tomography
